# Engineering the transmission efficiency of the noncyclic glyoxylate pathway for fumarate production in *Escherichia coli*

**DOI:** 10.1186/s13068-020-01771-3

**Published:** 2020-07-23

**Authors:** Xiulai Chen, Danlei Ma, Jia Liu, Qiuling Luo, Liming Liu

**Affiliations:** 1 State Key Laboratory of Food Science and Technology, Jiangnan University, 1800 Lihu Road, Wuxi, 214122 China; 2 Key Laboratory of Industrial Biotechnology, Ministry of Education, Jiangnan University, Wuxi, 214122 China; 3 International Joint Laboratory on Food Safety, Jiangnan University, Wuxi, 214122 China; 4 Wuxi Chenming Biotechnology Co. Ltd, Wuxi, 214100 China

**Keywords:** Fumarate, *Escherichia coli*, Pathway optimization, Transporter engineering, Metabolic engineering

## Abstract

**Background:**

Fumarate is a multifunctional dicarboxylic acid in the tricarboxylic acid cycle, but microbial engineering for fumarate production is limited by the transmission efficiency of its biosynthetic pathway.

**Results:**

Here, pathway engineering was used to construct the noncyclic glyoxylate pathway for fumarate production. To improve the transmission efficiency of intermediate metabolites, pathway optimization was conducted by fluctuating gene expression levels to identify potential bottlenecks and then remove them, resulting in a large increase in fumarate production from 8.7 to 16.2 g/L. To further enhance its transmission efficiency of targeted metabolites, transporter engineering was used by screening the C_4_-dicarboxylate transporters and then strengthening the capacity of fumarate export, leading to fumarate production up to 18.9 g/L. Finally, the engineered strain *E. coli* W3110△4-P_(H)_CAI_(H)_SC produced 22.4 g/L fumarate in a 5-L fed-batch bioreactor.

**Conclusions:**

In this study, we offered rational metabolic engineering and flux optimization strategies for efficient production of fumarate. These strategies have great potential in developing efficient microbial cell factories for production of high-value added chemicals.

## Background

Fumarate is a key intermediate in the tricarboxylic acid cycle (TCA) to link carbon and nitrogen metabolism, which has a variety of applications in many fields, such as food, pharmaceutical, bioplastic, and chemical industries [1]. Recently, fumarate is mainly produced through three major metabolic pathways, including the reductive TCA cycle [2], the oxidative TCA cycle [3], and the noncyclic glyoxylate cycle [4]. The maximum theoretical yield of fumarate is 2 mol/mol glucose in reductive TCA cycle, but its fumarate productivity is limited due to two reversible reactions catalyzed by malate dehydrogenase and fumarase [5]. Based on this reductive TCA cycle, fumarate productivity was increased to 0.30 g/L/h by combinatorially regulating the expression of phosphoenolpyruvate carboxykinase and formate dehydrogenase [5]. In addition, fumarate production via the oxidative TCA cycle provides a maximum theoretical yield of 1 mol/mol glucose due to its release of 2 CO_2_. Based on this oxidative TCA cycle, *Escherichia coli* CWF812 was able to produce 28.2 g/L with its productivity 0.448 g/L/h by deleting the *iclR*, *fumABC*, *arcA*, and *ptsG* genes and overexpressing the native *ppc* gene [6]. As for the noncyclic glyoxylate cycle, the maximum theoretical yield of fumarate is 1 mol/mol glucose. Although this pathway has shown its promising applications in improving the productivity of carboxylic acids [7], only few studies have focused on this pathway for fumarate production [8].

Two of the challenges in metabolically engineering the noncyclic glyoxylate cycle for fumarate production are how to identify and remove its potential bottlenecks and how to engineer and improve its transmission efficiency. Both challenges may benefit from the development of systems biology and synthetic biology. To identify and remove potential bottlenecks, several strategies have been developed, such as dynamic pathway analysis [9], X-omic technology [10], reverse metabolic engineering [11], in vitro metabolic engineering [12], and CRISPRi system [7]. To engineer and improve its transmission efficiency, many strategies have shown great potential, such as periplasmic engineering [13], mitochondrial engineering [14], DNA scaffold [15], protein scaffold [16], enzyme engineering [17], modular pathway engineering [18].

*Escherichia coli* is a well-established model microbe for industrial application, and it possesses many advantages. Many strategies in metabolic engineering and synthetic biology can be efficiently applied for genetic manipulation in *E. coli.* In addition, simple salt medium or cheap medium can be used for cell growth and the biosynthesis of high-value chemicals. Moreover, *E. coli* is particularly suitable for the production of carboxylic acids, such as lactate [19], pyruvate [20], and α-ketoglutarate [21]. Thus, *E. coli* is an attractive candidate for microbial engineering of C_4_-dicarboxylic acids production, due to the fact that it can provide a large amount of precursors (pyruvate or α-ketoglutarate) for the biosynthesis of C_4_-dicarboxylic acids such as fumarate [22], succinate [23], and malate [7]. Based on this observation, *E. coli* has a great potential in engineering the transmission efficiency of synthetic pathway to achieve high-level production of fumarate.

**Fig. 1:**
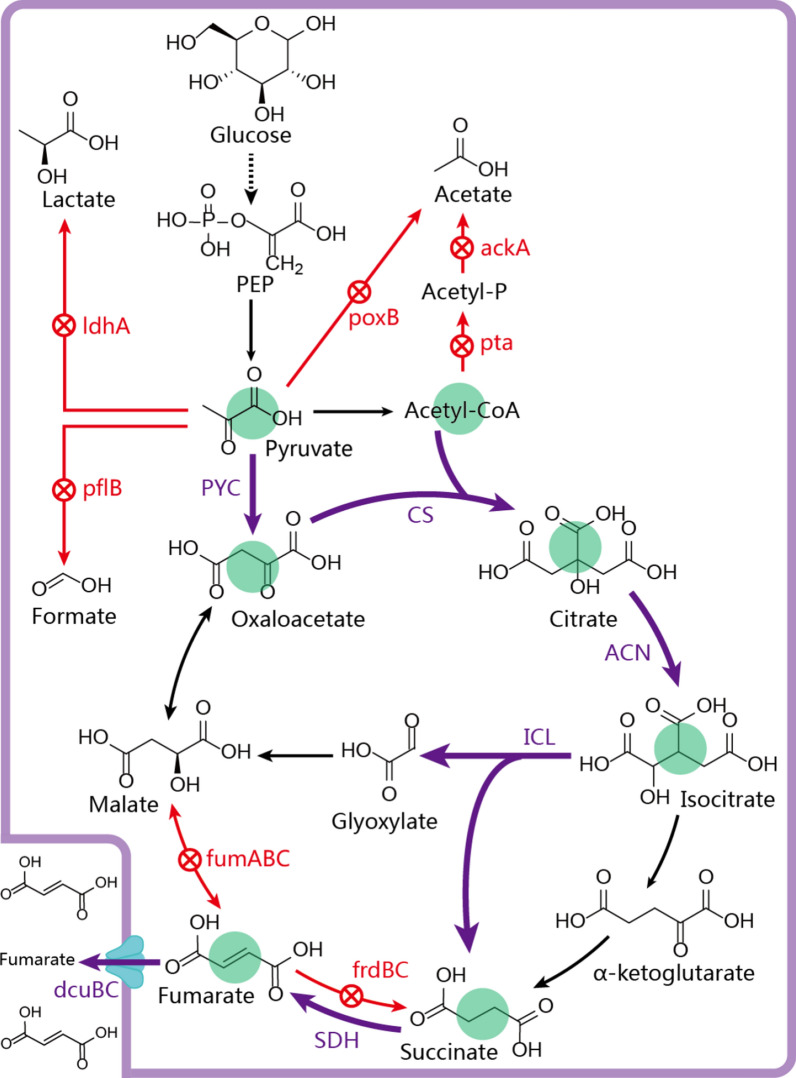
Major metabolic pathways for the formation of fumarate in *E. coli*. PEP: phosphoenolpyruvate; ldhA: lactate dehydrogenase; pflB: pyruvate formate lyase; poxB: pyruvate oxidase; pta: phosphotransacetylase; ackA: acetate kinase A; PYC: pyruvate carboxylase; CS: citrate synthase; ACN: aconitase; ICL: isocitrate lyase, SDH: succinate dehydrogenase; fumABC: fumarase; frdBC: fumarate reductase; dcuBC: the C4-dicarboxylate transporter

In this study, *Escherichia coli* W3110 was used as a host strain to rewire the noncyclic glyoxylate pathway for fumarate production (Fig. 1). Pathway optimization was conducted to identify and remove the potential bottlenecks, and then improve the transmission efficiency of intermediate metabolites. Further, transporter engineering was applied to enhance the transmission efficiency of targeted metabolites. Based on these strategies, the transmission efficiency of synthetic pathway was boosted, and the final engineered strain, *E. coli* W3110△4-P_(H)_CAI_(H)_SC, produced 22.4 g/L fumarate.

## Results

### Rewiring the noncyclic glyoxylate pathway for fumarate production

**Fig. 2:**
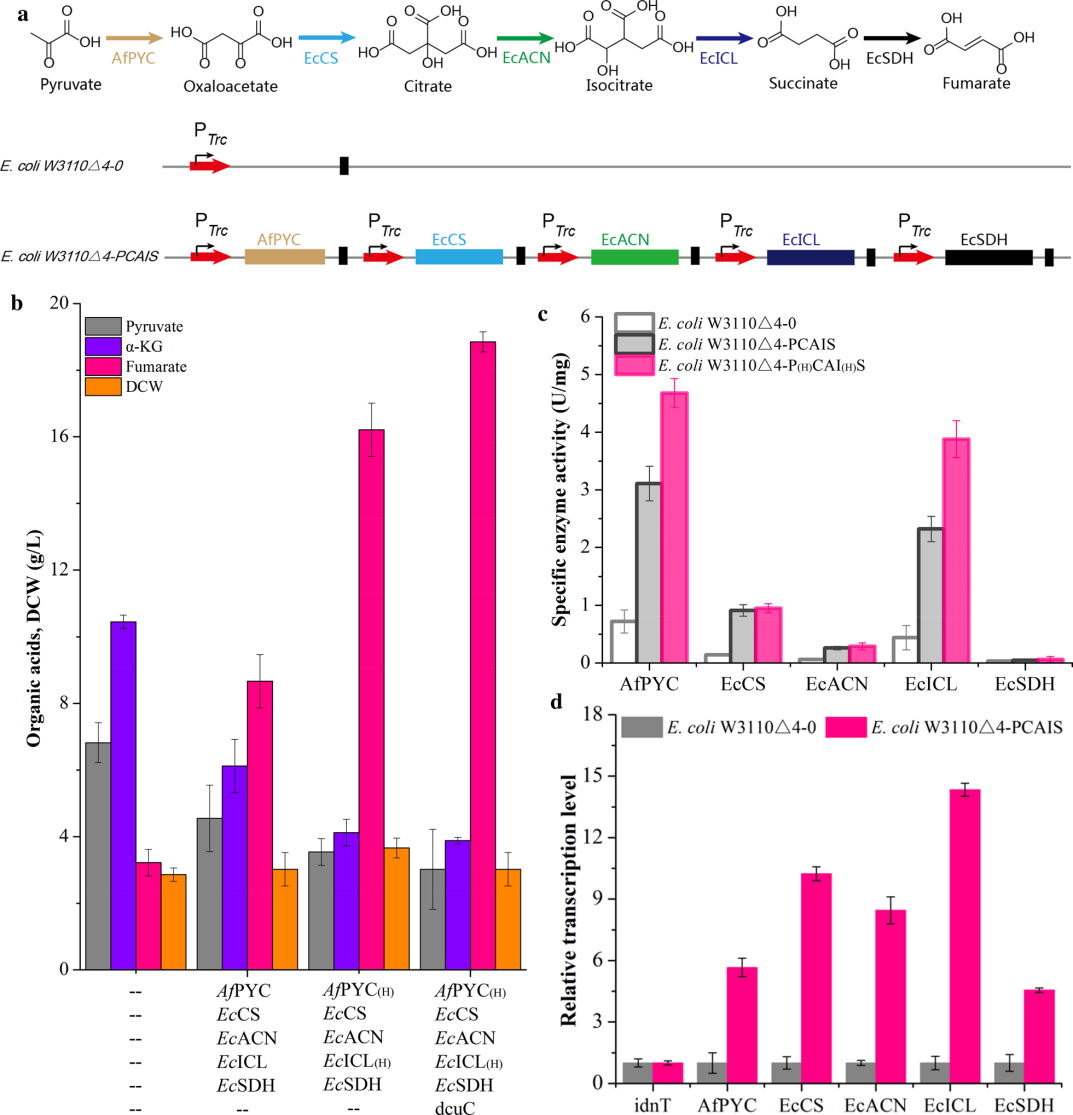
Constructing the noncyclic glyoxylate pathway for fumarate production. **a** Schematic representation of fumarate biosynthesis through the noncyclic glyoxylate pathway. **b** Effect of gene expression on the production of organic acids. **c** The specific activities of *Af*PYC, *Ec*CS, *Ec*ACN, *Ec*ICL, and *Ec*SDH. **d** The expression level of genes *Af*PYC, *Ec*CS, *Ec*ACN, *Ec*ICL, and *Ec*SDH. Error bars represent standard deviation from three biological replicates

*Escherichia coli* W3110△4 was constructed to produce pyruvate, L-malate, and α-ketoglutarate by deleting many genes including *ldhA*, *pflB*, *poxB*, *pta*, *ackA*, *frdBC*, and *fumABC* (Fig. 1) [24]. To further analyze its phenotypic characteristics, fermentation products of *E. coli* W3110△4 were measured, and we found that *E. coli* W3110△4 was able to produce 6.8 g/L pyruvate, 10.5 g/L α-ketoglutarate, and 3.2 g/L fumarate (Fig. 2b). Thus, *E. coli* W3110△4 was selected as a host strain for further metabolic engineering.

To rewire the noncyclic glyoxylate pathway for fumarate production, pyruvate carboxylase (*Af*PYC) [7], citrate synthase (*Ec*CS) [7], aconitase (*Ec*ACN) [7], isocitrate lyase (*Ec*ICL) [7], and succinate dehydrogenase (*Ec*SDH) [22] were selected and overexpressed in *E. coli* W3110△4 (Fig. 2a). By overexpressing these five enzymes simultaneously, the specific activities of *Af*PYC, *Ec*CS, *Ec*ACN, *Ec*ICL, and *Ec*SDH were increased by 3.3-, 5.5-, 3.3-, 4.3-, and 0.5-fold compared with these of *E. coli* W3110△4-0, respectively (Fig. 2c). In addition, the expression levels of *Af*PYC, *Ec*CS, *Ec*ACN, *Ec*ICL, and *Ec*SDH genes in strain *E. coli* W3110△4-PCAIS were all upregulated (Fig. 2d). Based on this, the final engineered strain *E. coli* W3110△4-PCAIS produced 8.7 g/L fumarate, which was 169.3% higher than that of strain *E. coli* W3110△4-0 (Fig. 2b). In addition, pyruvate and α-ketoglutarate were reduced by 33.3% and 41.4%, respectively, but DCW was increased by 5.6% (Fig. 2b). These results showed that the noncyclic glyoxylate pathway was successfully constructed, and could be used for fumarate production.

### Enhancing fumarate production by pathway optimization

To identify the potential bottlenecks in the noncyclic glyoxylate pathway, the expression level of individual enzymes was varied at different levels, while the remaining enzymes were all maintained at a fixed level. Based on this, we analyzed the effect of expression levels of every pathway enzyme on fumarate production, and the potential bottlenecks possibly showed a large variation range in fumarate production.

**Fig. 3:**
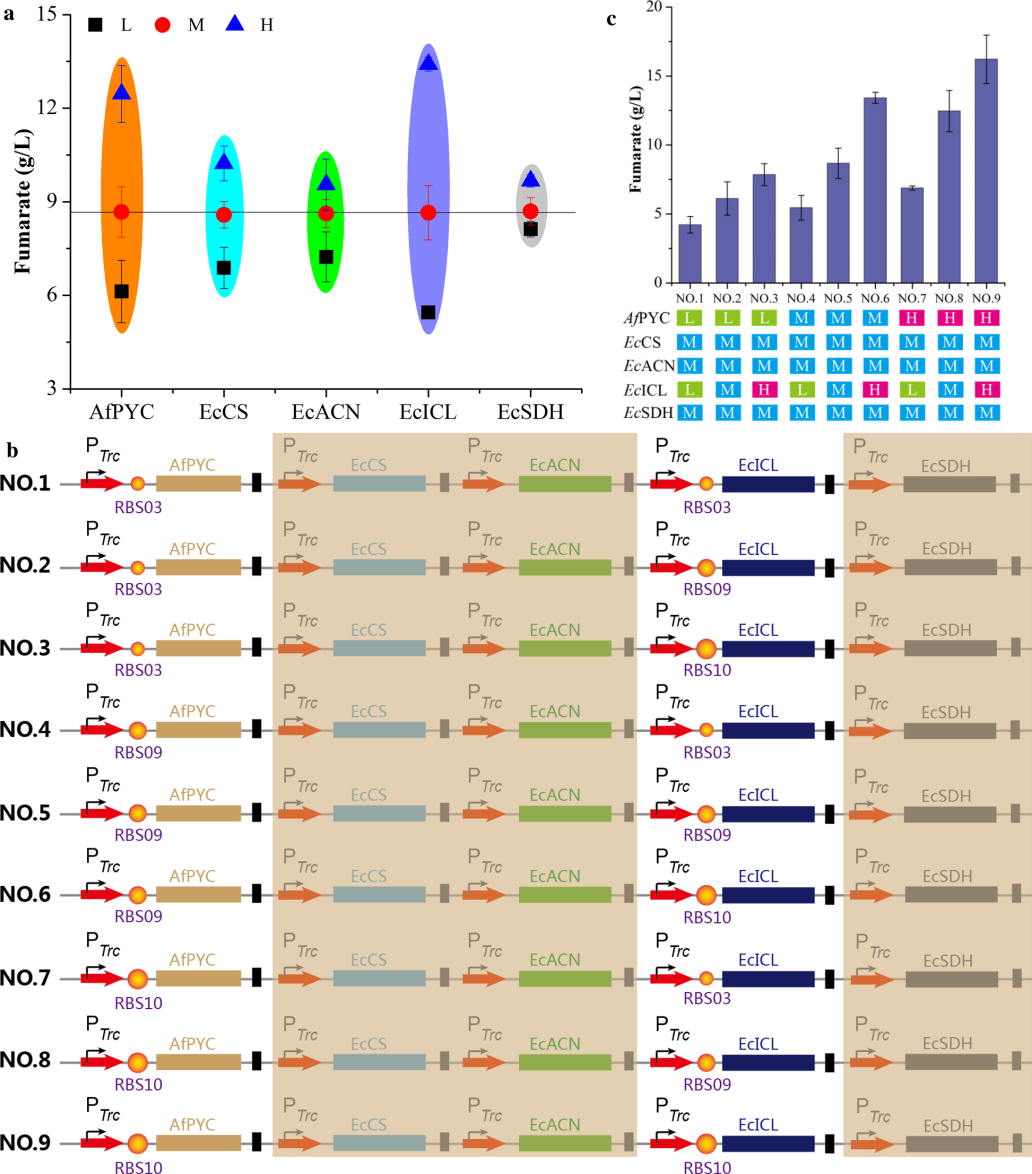
Optimizing the noncyclic glyoxylate pathway for fumarate production. **a** Effect of individual gene expression on fumarate production. **b** A series of *Af*PYC and *Ec*ICL expression cassettes were designed at different expression levels. **c** The concentrations of fumarate were achieved by different *Af*PYC and *Ec*ICL expression cassettes. Error bars represent standard deviation from three biological replicates

To demonstrate this idea, the strengths of gene expression were firstly set to three levels: high level (H) with RBS10, medium level (M) with RBS09, and low level (L) with RBS03 [25]. Then, many expression cassettes of *Af*PYC, *Ec*CS, *Ec*ACN, *Ec*ICL, and *Ec*SDH were introduced into *E. coli* W3110△4 to analyze its effects on fumarate production. When *Af*PYC expression was changed from low to high level, fumarate production was increased from 6.1 g/L to 12.5 g/L with variation range 103.6% (Fig. 3a). Similarly, with the increase of *Ec*ICL expression from low to high level, fumarate concentration was improved from 5.5 to 13.4 g/L with variation range 146.1% (Fig. 3a). However, the increasing strengths of *Ec*CS, *Ec*ACN, and *Ec*SDH expression showed a 48.7%, 32.1% and 19.1% increase in variation range of fumarate production, respectively (Fig. 3a). To sum up, *Af*PYC and *Ec*ICL expression led to a wider variation range in fumarate production than these of *Ec*CS, *Ec*ACN, and *Ec*SDH expression. These results possibly indicated that *Af*PYC and *Ec*ICL expression were the key nodes for further enhancing fumarate production.

**Fig. 4:**
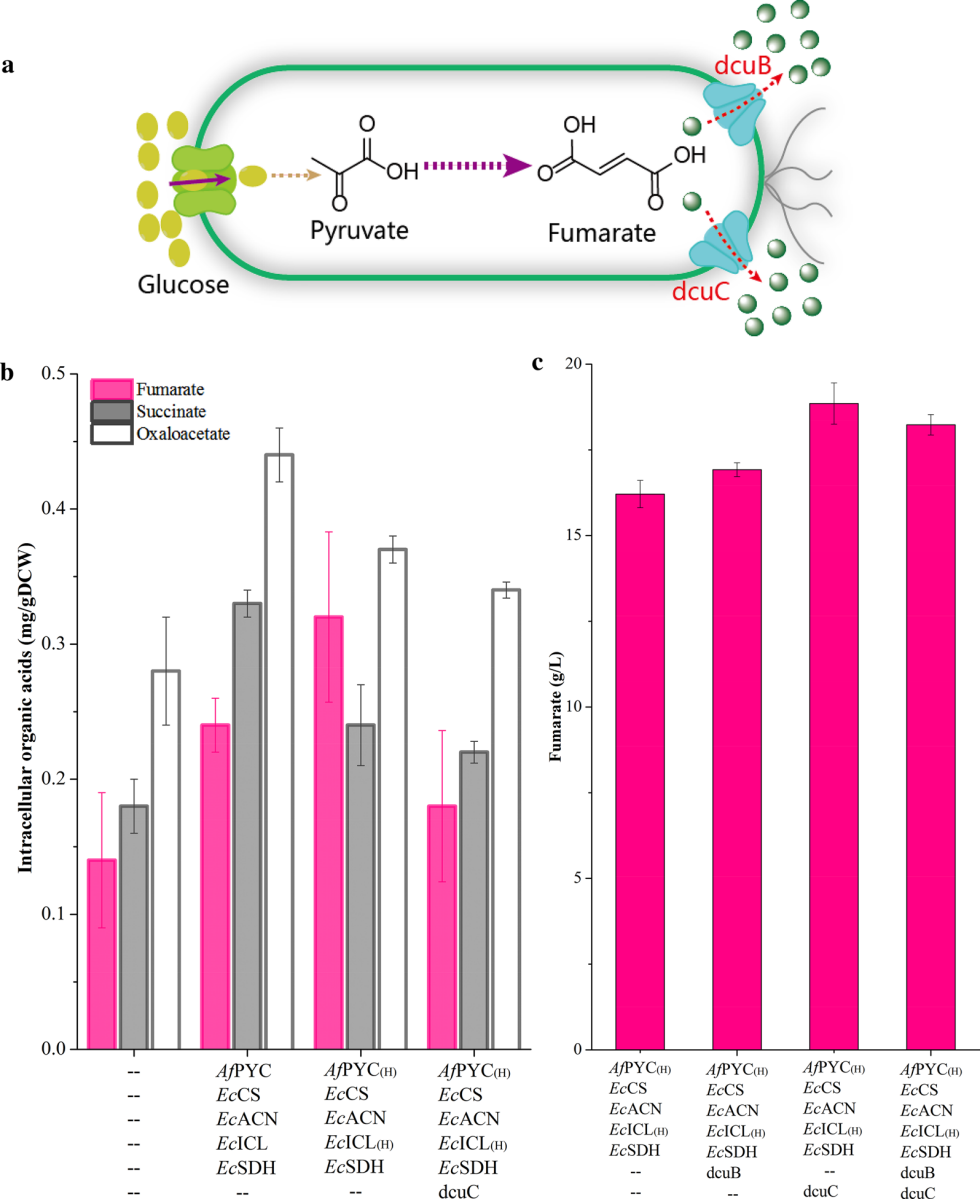
Improving fumarate production by transporter engineering. **a** Schematic representation of the C_4_-dicarboxylate transporters. **b** Effect of the C_4_-dicarboxylate transporters on the concentrations of intracellular fumarate, succinate, and oxaloacetate. **c** Effect of the C_4_-dicarboxylate transporters on fumarate production. Error bars represent standard deviation from three biological replicates

To further fine-tune the biosynthetic pathway for fumarate production, *Af*PYC and *Ec*ICL expression were optimized in *E. coli* W3110△4-PCAIS at three levels (H, M, and L) (Fig. 3b). Based on this, various expression cassettes of *Af*PYC and *Ec*ICL were introduced into the engineered *E. coli* to remove the potential bottlenecks, thus achieving the best distribution of metabolic flux for fumarate production. Finally, by controlling *Af*PYC and *Ec*ICL expression at a high level, fumarate production was increased to 16.2 g/L, which was 87.0% higher than that of strain *E. coli* W3110△4-PCAIS (Fig. 3c). At the same time, the specific activities of *Af*PYC and *Ec*ICL were increased by 50.5% and 67.2% compared with these of *E. coli* W3110△4-PCAIS, respectively (Fig. 2c). In addition, the intracellular succinate and oxaloacetate were decreased by 27.3% and 15.9%, respectively (Fig. 4b). However, the engineered strain *E. coli* W3110△4-P_(H)_CAI_(H)_S still accumulated 3.5 g/L pyruvate and 4.1 g/L α-ketoglutarate (Fig. 2b). Thus, there still existed other bottlenecks that need to be removed for further increasing production of fumarate.

### Improving fumarate production by transporter engineering

To further improve fumarate production, intracellular metabolites were analyzed for strains *E. coli* W3110△4-0 and *E. coli* W3110△4-P_(H)_CAI_(H)_S. The concentration of intracellular fumarate in strain *E. coli* W3110△4-P_(H)_CAI_(H)_S was increased by 128.6% compared with that of *E. coli* W3110△4-0 (Fig. 4b). Additionally, strain *E. coli* W3110△4-P_(H)_CAI_(H)_S showed a 21.2% increase in DCW compared with that of strain *E. coli* W3110△4-PCAIS (Fig. 2b). These results showed that the pyruvate flux was channelled not only to fumarate, but also to the TCA cycle, indicating that fumarate export probably needs to be engineered to transport fumarate more quickly.

The genes *dcuB* and *dcuC* encode the native C_4_-dicarboxylate transporters, which are used to export fumarate [26] (Fig. 4a). Thus, we tested the effects of *dcuB*, *dcuC*, and *dcuBC* on fumarate production, and the highest concentration of fumarate (18.9 g/L) was obtained with strain *E. coli* W3110△4-P_(H)_CAI_(H)_SC by overexpressing dcuC in strain *E. coli* W3110△4-P_(H)_CAI_(H)_S (Fig. 4c). This fumarate titer was 16.2% higher than that of strain *E. coli* W3110△4-P_(H)_CAI_(H)_S (Fig. 4c). In addition, DCW and the intracellular fumarate in strain *E. coli* W3110△4-P_(H)_CAI_(H)_SC were reduced by 17.5% and 43.8% compared to that of strain *E. coli* W3110△4-P_(H)_CAI_(H)_S, which was similar to that of the control strain *E. coli* W3110△4-0, respectively (Figs. 2b, 4b). Furthermore, pyruvate and α-ketoglutarate titers were decreased to 3.0 g/L and 3.9 g/L, which were lower than these of strains *E. coli* W3110△4-0 and *E. coli* W3110△4-P_(H)_CAI_(H)_S, respectively (Fig. 2b). These results indicated that the C_4_-dicarboxylate transporter was efficient for fumarate export.

### Production of fumarate in a 5-L bioreactor

**Fig. 5:**
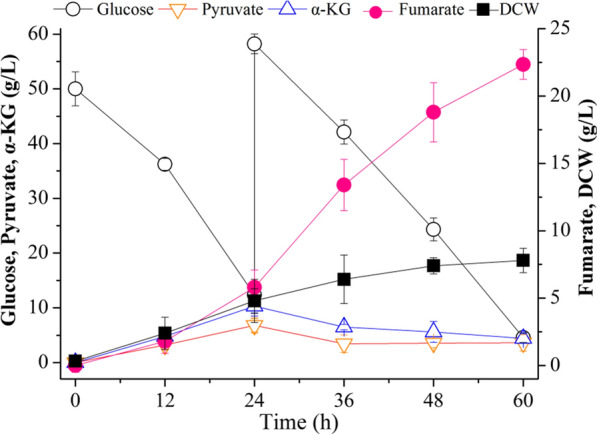
Production of fumarate by strain *E. coli* W3110△4-P_(H)_CAI_(H)_SC in a 5-L fed-batch bioreactor. Error bars represent standard deviation from three biological replicates

Fumarate production with the optimized strain *E. coli* W3110△4-P_(H)_CAI_(H)_SC was tested in a 5-L fed-batch bioreactor. In this process, fumarate was accumulated gradually, and the maximal concentration of fumarate was up to 22.4 g/L at 60 h, which was 18.6% higher than that in shake flasks (Fig. 5). With the accumulation of fumarate, glucose was consumed rapidly, and nearly depleted at 60 h (Fig. 5). In addition, cell growth was increased continuously, and the maximal DCW was up to 7.8 g/L at 60 h, which showed a 158.3% increase compared to that in shake flasks (Fig. 5). These results indicated that the final strain *E. coli* W3110△4-P_(H)_CAI_(H)_SC has great potential for efficient production of fumarate in fermentation.

## Discussion

Metabolic engineering is conducted to rewire the complete noncyclic glyoxylate pathway for fumarate production. Recently, five metabolic engineering strategies have been developed to enhance production of fumarate: reconstructing synthetic pathway, such as the reductive TCA cycle [2], the oxidative TCA cycle [3], the noncyclic glyoxylate cycle [4], the urea cycle and the purine nucleotide cycle [27, 28]; eliminating byproducts formation [27], such as lactate, acetate, formate, malate, and succinate; optimizing oxidation and reduction levels [5]; modifying glucose transport system [8]; regulating C_4_-dicarboxylate transporter [26]. These results indicated that fumarate production has been improved by metabolic engineering strategies. However, in these previous studies, fumarate production was enhanced by constructing the partial metabolic pathway, rather than the complete metabolic pathway, thus resulting in the transmission inefficiency of these synthetic pathways. Among these studies, when the biosynthetic pathway was reconstructed in the evolved mutant *E. coli* E2 by combining the oxidative TCA cycle and the glyoxylate cycle partially, fumarate production showed a large increase up to 41.5 g/L [4]. The main differences between this previous study and our study are that (i) we constructed the complete noncyclic glyoxylate pathway to produce fumarate, rather than the partial or combined metabolic pathways; (ii) we rationally identified and removed the potential bottlenecks in the complete noncyclic glyoxylate pathway, rather than irrationally redirecting metabolic flux; (iii) we definitely optimized the balance of metabolic pathway, rather than indefinitely distributing carbon flux. To sum up, although fumarate production with *E. coli* W3110△4-P_(H)_CAI_(H)_SC in our study was lower than that of *E. coli* EF02 (pSCppc) in previous study, we offered rational metabolic engineering and flux optimization strategies for efficient production of fumarate. These strategies have great potential in developing efficient microbial cell factories for production of high-value added chemicals.

Pathway optimization represents one significant step in identifying and removing the potential bottlenecks to improve the transmission efficiency of biosynthetic pathway. To improve the transmission efficiency of intermediate metabolites, the partial noncyclic glyoxylate pathway was constructed by replacing the native PEP-dependent PTSG system with the PEP-independent galactose translocation system, overexpressing phosphoenolpyruvate carboxylase (PPC) and acetyl-CoA synthase, and deleting malate dehydrogenase, fumarate reductase, and fumarase [8]. The final concentration of fumarate (1.53 g/g dry cell weight) was increased by 50% compared with the parental strain. Further, the partial noncyclic glyoxylate pathway was enhanced by overexpressing PPC, succinate dehydrogenase complex, and citrate synthase (CS) [22]. After that, the expression levels of PPC and CS were optimized, and the final strain *E. coli* CWF4NS (pSynPC39) produced 25.5 g/L fumarate with its productivity of 0.35 g/L/h. These results showed that fumarate production could be improved by engineering the partial noncyclic glyoxylate pathway. However, fumarate production might be further increased by constructing and optimizing the complete noncyclic glyoxylate pathway to reinforce its transmission efficiency. In our study, pathway optimization was successfully applied to identify and remove the potential bottlenecks in the complete noncyclic glyoxylate pathway, and thus improve its transmission efficiency. The optimized strain *E. coli* W3110△4-P_(H)_CAI_(H)_SC was able to produce 22.4 g/L fumarate with its productivity of 0.37 g/L/h. These results indicated that pathway optimization could significantly increase the transmission efficiency of intermediate metabolites. This strategy not only could improve the transmission efficiency of intermediate metabolites, but also could reduce the loss of carbon flux. The increased transmission efficiency of biosynthetic pathway for fumarate production is possibly due to the balanced substrate channeling: (i) the local concentration of intermediates can appropriately meet the need of pathway enzymes by fine-tuning gene expression to balance biosynthetic pathway; (ii) the carbon flux of intermediates is efficiently concentrated on producing fumarate by preventing the competing pathways to reduce carbon loss; (iii) the feedback inhibition of intermediates is successfully circumvented by converting these inhibitors into another valid intermediate rapidly.

Transporter engineering is another useful step in enhancing the transmission efficiency of biosynthetic pathway. Transporters can be rationally regulated to recognize and transport targeted metabolites between inside and outside the cell, which has shown great promise in reducing toxicity and increasing productivity [29]. Recent strategies mainly center on two types: (i) ABC transporters mainly contain an exporter for pumping out the final products [30] and an importer for improving the absorption of substances [31]; (ii) secondary efflux pumps are able to excrete toxic compounds actively [32]. Based on this, to improve the transmission efficiency of targeted metabolites, the C4-dicarboxylate transporters (dcuBC) were overexpressed in *E. coli* ABCDIA [26], in which the biosynthetic pathway for fumarate production was constructed by combining the urea cycle and the glyoxylate cycle partially [27]. The resulting strain *E. coli* A-dcuB-Ec only produced 9.42 g/L fumarate with its productivity 0.19 g/L/h [26], possibly due to the fact that there is no complete or efficient biosynthetic pathway to supply enough fumarate to meet the need of transporters, thus reducing the transmission efficiency of targeted metabolites. In our study, transporter engineering was carried out in the engineered *E. coli* strain harboring the complete noncyclic glyoxylate pathway for fumarate production. Based on this, there is enough intracellular fumarate to be transported outside the cell by dcuBC, and the productivity of fumarate was increased to 0.37 g/L/h. These results indicated that transporter engineering could largely increase the transmission efficiency of targeted metabolites. This strategy not only could prevent fumarate accumulation in the intracellular space, but also could alleviate the toxicity of intracellular fumarate. The improved transmission efficiency of biosynthetic pathway for fumarate production is also possibly due to the fact that dcuBC are efficient for exporting fumarate to prevent self-poisoning and reduce feedback inhibition, thus ultimately realizing maximum production of fumarate.

## Conclusions

In this study, the noncyclic glyoxylate pathway was successfully constructed for fumarate synthesis. The transmission efficiency of intermediate metabolites was enhanced by optimizing the expression levels of pathway enzymes. Further, the transmission efficiency of targeted metabolites was improved by strengthening the C_4_-dicarboxylate transporters. Based on these strategies, fumarate production with strain *E. coli* W3110△4-P_(H)_CAI_(H)_SC was increased from 3.2 to 22.4 g/L. These strategies have great application potential in developing efficient microbial cell factories for production of high-value added chemicals.

## Materials and methods

### Strains and plasmids

*Escherichia coli* W3110△*ldhA*△*poxB*△*pflB*△*pta*-*ackA*△*frdBC*△*fumB*△*fumAC* (*E. coli* W3110△4) was applied as host strain for overexpressing key genes [24]. The engineered *E. coli* strains for fumarate production were all derived from *E. coli* W3110△4. *E. coli* JM109 and plasmid pETM6R1 [25] were used for constructing key plasmids. All strains and plasmids are listed in Additional file 1: Table S1.

### DNA manipulation

Gibson Assembly was applied for constructing key plasmids according to the protocol of Gibson Assembly Cloning Kit (NEB), respectively. Pyruvate carboxylase gene from *Aspergillus flavus* (*Af*PYC, AFLA_112120) was amplified by PCR using plasmids pTrcHisA-*Af*PYC as template [7]. Citrate synthase (*Ec*CS, b0720), aconitase (*Ec*ACN, b0118) and isocitrate lyase (*Ec*ICL, b4015) genes from *E. coli* were amplified from plasmids pET28a-*Ec*CS, pET28a-*Ec*ACN, and pET28a-*Ec*ICL, respectively [7]. Succinate dehydrogenase (SDH, b0723) [22], the C4-dicarboxylate transporters dcuC (b0621) and dcuB (b4123) genes were PCR-amplified from the genome of *E. coli* W3110 [26].

### Medium

LB medium used for seed culture: 5 g/L yeast extract, 10 g/L peptone, 5 g/L NaCl. Ampicillin (100 mg/mL) was added to LB medium appropriately when needed.

Modified M9 minimal medium used for fermentation: 50 g/L glucose, 10 g/L yeast extract, 0.5 g/L NH_4_Cl, 1 g/L citrate, 3 g/L NaHCO_3_, 7.52 g/L Na_2_HPO_4_-2H_2_O, 3 g/L KH_2_PO4, 0.5 g/L NaCl, 0.246 g/L MgSO_4_, 0.044 g/L CaCl_2_, 1 μg/L biotin, 1 μg/L thiamin, and 1 mL trace element solution (2.4 g/L FeCl_3_-6H_2_O, 0.3 g/L CoCl_2_-6H_2_O, 0.3 g/L CuCl_2_, 0.3 g/L ZnCl_2_-4H_2_O, 0.3 g/L NaMnO_4_, 0.075 g/L H_3_BO_3_, 0.5 g/L MnCl_2_-4H_2_O, dissolve in 0.12 M HCl). Ampicillin (100 mg/mL) and IPTG (0.4 mmol/L) were added appropriately when needed.

### Culture conditions

The seed culture was cultivated at 37 °C for 12 h with rotation at 200 rpm in a 250-mL flask containing 25 mL LB medium. After that, the broth was centrifuged to discard supernatant liquid, and then fresh M9 medium was used to suspend the pellet. Next, the cell suspension was spread equally across 500-mL flasks with 50 mL fresh M9 medium with an initial biomass OD_600_ = 0.5. This cell culture was buffered by 30 g/L CaCO_3_, and fermented at 37 °C for 60 h with rotation at 200 rpm. 50 g/L glucose was fed at 24 h.

Fermentation was conducted in a 5-L fed-batch bioreactor containing 2.5 L M9 medium with an initial biomass OD_600_ = 0.5 at 37 °C for 60 h. Agitation speed and aeration rate were controlled at 200 rpm and 1.0 vvm, respectively. Culture pH was controlled at 7.0 using 20% (w/v) Na_2_CO_3_. 50 g/L glucose was fed at 24 h.

### Analytical methods

The optical density at 600 nm (OD_600_) was assayed by a spectrophotometer (1OD_600_ = 0.33 g/L DCW (Dry Cell Weight)). Glucose concentration was quantified by a biosensor SBA-90 [24]. The concentration of organic acids was detected by high-performance liquid chromatography (HPLC) [24].

Intracellular metabolites were extracted by freezing-thawing in methanol [33]. The intracellular fumarate, succinate, and oxaloacetate were determined by HPLC according to the procedure described in previous reports [24].

### Transcriptional analysis

Total RNA was extracted by the RNAprep pure Kit (TIANGEN), and reverse transcription was conducted for cDNA synthesis as described in the protocol of Reverse Transcription Kit (Takara). Real-time quantitative PCR was carried out according to [34]. L-Idonate/5-ketogluconate/gluconate transporter gene (*idnT*) was used as reference gene.

### Enzyme activity assays

Pyruvate carboxylase (PYC) was assayed as reported by [35]. Citrate synthase (CS) was analyzed as described by [36]. Aconitase (ACN) was detected according to [37]. Isocitrate lyase (ICL) was determined as previously described methods [7]. Succinate dehydrogenase (SDH) was measured by the method of [38].

## Supplementary information

**Additional file 1: Table S1.** Strains and plasmids used in this study.

## Data Availability

The dataset supporting the conclusions of this article is included in the article.

## Abbreviations

PEP: Phosphoenolpyruvate
ldhA: Lactate dehydrogenase
pflB: Pyruvate formate lyase
poxB: Pyruvate oxidase
pta: Phosphotransacetylase
ackA: Acetate kinase A
PYC: Pyruvate carboxylase
CS: Citrate synthase
ACN: Aconitase
ICL: Isocitrate lyase
SDH: Succinate dehydrogenase
fumABC: Fumarase
frdBC: Fumarate reductase
dcuBC: The C4-dicarboxylate transporter

## References

[1] Zhou Y, Nie K, Zhang X, Liu S, Wang M, Deng L, Wang F, Tan T (2014). Production of fumaric acid from biodiesel-derived crude glycerol by *Rhizopus arrhizus*. Bioresour Technol.

[2] Xu G, Liu L, Chen J (2012). Reconstruction of cytosolic fumaric acid biosynthetic pathways in *Saccharomyces cerevisiae*. Microb Cell Fact.

[3] Chen X, Dong X, Wang Y, Zhao Z, Liu L (2015). Mitochondrial engineering of the TCA cycle for fumarate production. Metab Eng.

[4] Li N, Zhang B, Wang Z, Tang YJ, Chen T, Zhao X (2014). Engineering *Escherichia coli* for fumaric acid production from glycerol. Bioresour Technol.

[5] Chen X, Li Y, Tong T, Liu L (2019). Spatial modulation and cofactor engineering of key pathway enzymes for fumarate production in *Candida glabrata*. Biotechnol Bioeng.

[6] Song CW, Kim DI, Choi S, Jang JW, Lee SY (2013). Metabolic engineering of *Escherichia coli* for the production of fumaric acid. Biotechnol Bioeng.

[7] Gao C, Wang S, Hu G, Guo L, Chen X, Xu P, Liu L (2018). Engineering *Escherichia coli* for malate production by integrating modular pathway characterization with CRISPRi-guided multiplexed metabolic tuning. Biotechnol Bioeng.

[8] Liu H, Song R, Liang Y, Zhang T, Deng L, Wang F, Tan T (2018). Genetic manipulation of *Escherichia coli* central carbon metabolism for efficient production of fumaric acid. Bioresour Technol.

[9] Liu Y, Link H, Liu L, Du G, Chen J, Sauer U (2016). A dynamic pathway analysis approach reveals a limiting futile cycle in *N*-acetylglucosamine overproducing *Bacillus subtilis*. Nat Commun.

[10] Link H, Fuhrer T, Gerosa L, Zamboni N, Sauer U (2015). Real-time metabolome profiling of the metabolic switch between starvation and growth. Nat Methods.

[11] Bailey JE, Sburlati A, Hatzimanikatis V, Lee K, Renner WA, Tsai PS (2002). Inverse metabolic engineering: a strategy for directed genetic engineering of useful phenotypes. Biotechnol Bioeng.

[12] Opgenorth PH, Korman TP, Bowie JU (2016). A synthetic biochemistry module for production of bio-based chemicals from glucose. Nat Chem Biol.

[13] Guo L, Zhang F, Zhang C, Hu G, Gao C, Chen X, Liu L (2018). Enhancement of malate production through engineering of the periplasmic rTCA pathway in *Escherichia coli*. Biotechnol Bioeng.

[14] Avalos JL, Fink GR, Stephanopoulos G (2013). Compartmentalization of metabolic pathways in yeast mitochondria improves the production of branched-chain alcohols. Nat Biotechnol.

[15] Conrado RJ, Wu GC, Boock JT, Xu H, Chen SY, Lebar T, Turnsek J, Tomsic N, Avbelj M, Gaber R, Koprivnjak T, Mori J, Glavnik V, Vovk I, Bencina M, Hodnik V, Anderluh G, Dueber JE, Jerala R, DeLisa MP (2012). DNA-guided assembly of biosynthetic pathways promotes improved catalytic efficiency. Nucleic Acids Res.

[16] Dueber JE, Wu GC, Malmirchegini GR, Moon TS, Petzold CJ, Ullal AV, Prather KL, Keasling JD (2009). Synthetic protein scaffolds provide modular control over metabolic flux. Nat Biotechnol.

[17] Zhou YJ, Gao W, Rong Q, Jin G, Chu H, Liu W, Yang W, Zhu Z, Li G, Zhu G, Huang L, Zhao ZK (2012). Modular pathway engineering of diterpenoid synthases and the mevalonic acid pathway for miltiradiene production. J Am Chem Soc.

[18] Ajikumar PK, Xiao WH, Tyo KE, Wang Y, Simeon F, Leonard E, Mucha O, Phon TH, Pfeifer B, Stephanopoulos G (2010). Isoprenoid pathway optimization for Taxol precursor overproduction in *Escherichia coli*. Science.

[19] Mazumdar S, Blankschien MD, Clomburg JM, Gonzalez R (2013). Efficient synthesis of L-lactic acid from glycerol by metabolically engineered *Escherichia coli*. Microb Cell Fact.

[20] Causey TB, Shanmugam KT, Yomano LP, Ingram LO (2004). Engineering *Escherichia coli* for efficient conversion of glucose to pyruvate. Proc Natl Acad Sci USA.

[21] Chen X, Dong X, Liu J, Luo Q, Liu L (2020). Pathway engineering of *Escherichia coli* for α-ketoglutaric acid production. Biotechnol Bioeng.

[22] Song CW, Lee SY (2015). Combining rational metabolic engineering and flux optimization strategies for efficient production of fumaric acid. Appl Microbiol Biotechnol.

[23] Lin H, Bennett GN, San KY (2005). Metabolic engineering of aerobic succinate production systems in *Escherichia coli* to improve process productivity and achieve the maximum theoretical succinate yield. Metab Eng.

[24] Dong X, Chen X, Qian Y, Wang Y, Wang L, Qiao W, Liu L (2017). Metabolic engineering of *Escherichia coli* W3110 to produce L-malate. Biotechnol Bioeng.

[25] Zhang Q, Yao R, Chen X, Liu L, Xu S, Chen J, Wu J (2018). Enhancing fructosylated chondroitin production in *Escherichia coli* K4 by balancing the UDP-precursors. Metab Eng.

[26] Zhang T, Song RR, Wang M, Deng L, Fan LH, Wang F (2017). Regulating C4-dicarboxylate transporters for improving fumaric acid production. Rsc Adv.

[27] Zhang T, Wang Z, Deng L, Tan T, Wang F, Yan Y (2015). Pull-in urea cycle for the production of fumaric acid in *Escherichia coli*. Appl Microbiol Biotechnol.

[28] Chen X, Wu J, Song W, Zhang L, Wang H, Liu L (2015). Fumaric acid production by *Torulopsis glabrata*: engineering the urea cycle and the purine nucleotide cycle. Biotechnol Bioeng.

[29] Huffer S, Roche CM, Blanch HW, Clark DS (2012). *Escherichia coli* for biofuel production: bridging the gap from promise to practice. Trends Biotechnol.

[30] Qiu J, Zhuo Y, Zhu D, Zhou X, Zhang L, Bai L, Deng Z (2011). Overexpression of the ABC transporter AvtAB increases avermectin production in *Streptomyces avermitilis*. Appl Microbiol Biotechnol.

[31] Davidson AL, Dassa E, Orelle C, Chen J (2008). Structure, function, and evolution of bacterial ATP-binding cassette systems. Microbiol Mol Biol Rev.

[32] Higgins CF (2007). Multiple molecular mechanisms for multidrug resistance transporters. Nature.

[33] Canelas AB, ten Pierick A, Ras C, Seifar RM, van Dam JC, van Gulik WM, Heijnen JJ (2009). Quantitative evaluation of intracellular metabolite extraction techniques for yeast metabolomics. Anal Chem.

[34] Wu Q, Yang A, Zou W, Duan Z, Liu J, Chen J, Liu L (2013). Transcriptional engineering of *Escherichia coli* K4 for fructosylated chondroitin production. Biotechnol Prog.

[35] Zelle RM, de Hulster E, van Winden WA, de Waard P, Dijkema C, Winkler AA, Geertman JM, van Dijken JP, Pronk JT, van Maris AJ (2008). Malic acid production by *Saccharomyces cerevisiae*: engineering of pyruvate carboxylation, oxaloacetate reduction, and malate export. Appl Environ Microbiol.

[36] Vuoristo KS, Mars AE, Sangra JV, Springer J, Eggink G, Sanders JP, Weusthuis RA (2015). Metabolic engineering of itaconate production in *Escherichia coli*. Appl Microbiol Biotechnol.

[37] Baumgart M, Bott M (2011). Biochemical characterisation of aconitase from *Corynebacterium glutamicum*. J Biotechnol.

[38] Kubo Y, Takagi H, Nakamori S (2000). Effect of gene disruption of succinate dehydrogenase on succinate production in a sake yeast strain. J Biosci Bioeng.

