# Supplementary material for: Engineering the transmission efficiency of the noncyclic glyoxylate pathway for fumarate production in Escherichia coli
Source: Biotechnol Biofuels. 2020 Jul 23;13:132. doi: 10.1186/s13068-020-01771-3 (PMC7379832; doi:10.1186/s13068-020-01771-3)
Supplement: Supplementary file 1 — Additional file 1: Table S1. Strains and plasmids used in this study. [file 13068_2020_1771_MOESM1_ESM.doc]

Engineering the transmission efficiency of the noncyclic glyoxylate pathway for fumarate production in *Escherichia coli*

Xiulai Chen^a,b,c^, Danlei Ma^a,b,c^, Jia Liu^a,b,c^, Qiuling Luo^a,d^, and Liming Liu^a,b,c^*

^a^ State Key Laboratory of Food Science and Technology, Jiangnan University, Wuxi 214122, China;

^b^ Key Laboratory of Industrial Biotechnology, Ministry of Education, Jiangnan University, Wuxi 214122, China;

^c^ International Joint Laboratory on Food Safety, Jiangnan University, Wuxi 214122, China;

^d^ Wuxi Chenming Biotechnology Co. Ltd, Wuxi 214100, China

*Corresponding author at: State Key Laboratory of Food Science and Technology, Jiangnan University, 1800 Lihu Road, Wuxi 214122, China. Fax: +86-510-85197875

*E-mail address*: [**mingll@jiangnan.edu.cn**](mailto:mingll@jiangnan.edu.cn) (Liming Liu)

This doc file includes:

Supplementary Tables S1

**Table S1** Strains and plasmids used in this study.

| **Strains and** **plasmids** | **Relevant characteristics** | **References** |
| --- | --- | --- |
| **Strains** |  |  |
| *E. coli* W3110 | F^-^λ^-^rph-1 INV(rrnD, rrnE) | CGSC |
| *E. coli* W3110△4 | *E. coli* W3110△*ldhA*△*poxB*△*pflB*△*pta-ackA*△*frdBC*△*fumB*△*fumAC* |  |
| *E. coli* W3110△4-0 | *E. coli* W3110△*ldhA*△*pflB*△*poxB*△*pta-ackA*△*frdBC*△*fumB*△*fumAC*  (pETM6R1) | This study |
| *E. coli* W3110△4-PCAIS | *E. coli* W3110△*ldhA*△*poxB*△*pflB*△*pta-ackA*△*frdBC*△*fumB*△*fumAC*  (pETM6R1-*Af*PYC-*Ec*CS-*Ec*ACN-*Ec*ICL-*Ec*SDH) | This study |
| *E. coli* W3110△4-P_(L)_CAIS | *E. coli* W3110△*ldhA*△*poxB*△*pflB*△*pta-ackA*△*frdBC*△*fumB*△*fumAC*  (pETM6R1-_(RBS03)_*Af*PYC-*Ec*CS-*Ec*ACN-*Ec*ICL-*Ec*SDH) | This study |
| *E. coli* W3110△4-P_(M)_CAIS | *E. coli* W3110△*ldhA*△*poxB*△*pflB*△*pta-ackA*△*frdBC*△*fumB*△*fumAC*  (pETM6R1-_(RBS09)_*Af*PYC-*Ec*CS-*Ec*ACN-*Ec*ICL-*Ec*SDH) | This study |
| *E. coli* W3110△4-P_(H)_CAIS | *E. coli* W3110△*ldhA*△*poxB*△*pflB*△*pta-ackA*△*frdBC*△*fumB*△*fumAC*  (pETM6R1-_(RBS10)_*Af*PYC-*Ec*CS-*Ec*ACN-*Ec*ICL-*Ec*SDH) | This study |
| *E. coli* W3110△4-PC_(L)_AIS | *E. coli* W3110△*ldhA*△*poxB*△*pflB*△*pta-ackA*△*frdBC*△*fumB*△*fumAC*  (pETM6R1-*Af*PYC-_(RBS03)_*Ec*CS-*Ec*ACN-*Ec*ICL-*Ec*SDH) | This study |
| *E. coli* W3110△4-PC_(M)_AIS | *E. coli* W3110△*ldhA*△*poxB*△*pflB*△*pta-ackA*△*frdBC*△*fumB*△*fumAC*  (pETM6R1-*Af*PYC-_(RBS09)_*Ec*CS-*Ec*ACN-*Ec*ICL-*Ec*SDH) | This study |
| *E. coli* W3110△4-PC_(H)_AIS | *E. coli* W3110△*ldhA*△*poxB*△*pflB*△*pta-ackA*△*frdBC*△*fumB*△*fumAC*  (pETM6R1-*Af*PYC-_(RBS10)_*Ec*CS-*Ec*ACN-*Ec*ICL-*Ec*SDH) | This study |
| *E. coli* W3110△4-PCA_(L)_IS | *E. coli* W3110△*ldhA*△*poxB*△*pflB*△*pta-ackA*△*frdBC*△*fumB*△*fumAC*  (pETM6R1-*Af*PYC-*Ec*CS-_(RBS03)_*Ec*ACN-*Ec*ICL-*Ec*SDH) | This study |
| *E. coli* W3110△4-PCA_(M)_IS | *E. coli* W3110△*ldhA*△*poxB*△*pflB*△*pta-ackA*△*frdBC*△*fumB*△*fumAC*  (pETM6R1-*Af*PYC-*Ec*CS-_(RBS09)_*Ec*ACN-*Ec*ICL-*Ec*SDH) | This study |
| *E. coli* W3110△4-PCA_(H)_IS | *E. coli* W3110△*ldhA*△*poxB*△*pflB*△*pta-ackA*△*frdBC*△*fumB*△*fumAC*  (pETM6R1-*Af*PYC-*Ec*CS-_(RBS10)_*Ec*ACN-*Ec*ICL-*Ec*SDH) | This study |
| *E. coli* W3110△4-PCAI_(L)_S | *E. coli* W3110△*ldhA*△*poxB*△*pflB*△*pta-ackA*△*frdBC*△*fumB*△*fumAC*  (pETM6R1-*Af*PYC-*Ec*CS-*Ec*ACN-_(RBS03)_*Ec*ICL-*Ec*SDH) | This study |
| *E. coli* W3110△4-PCAI_(M)_S | *E. coli* W3110△*ldhA*△*poxB*△*pflB*△*pta-ackA*△*frdBC*△*fumB*△*fumAC*  (pETM6R1-*Af*PYC-*Ec*CS-*Ec*ACN-_(RBS09)_*Ec*ICL-*Ec*SDH) | This study |
| *E. coli* W3110△4-PCAI_(H)_S | *E. coli* W3110△*ldhA*△*poxB*△*pflB*△*pta-ackA*△*frdBC*△*fumB*△*fumAC*  (pETM6R1-*Af*PYC-*Ec*CS-*Ec*ACN-_(RBS10)_*Ec*ICL-*Ec*SDH) | This study |
| *E. coli* W3110△4-PCAIS_(L)_ | *E. coli* W3110△*ldhA*△*poxB*△*pflB*△*pta-ackA*△*frdBC*△*fumB*△*fumAC*  (pETM6R1-*Af*PYC-*Ec*CS-*Ec*ACN-*Ec*ICL-_(RBS03)_*Ec*SDH) | This study |
| *E. coli* W3110△4-PCAIS_(M)_ | *E. coli* W3110△*ldhA*△*poxB*△*pflB*△*pta-ackA*△*frdBC*△*fumB*△*fumAC*  (pETM6R1-*Af*PYC-*Ec*CS-*Ec*ACN-*Ec*ICL-_(RBS09)_*Ec*SDH) | This study |
| *E. coli* W3110△4-PCAIS_(H)_ | *E. coli* W3110△*ldhA*△*poxB*△*pflB*△*pta-ackA*△*frdBC*△*fumB*△*fumAC*  (pETM6R1-*Af*PYC-*Ec*CS-*Ec*ACN-*Ec*ICL-_(RBS10)_*Ec*SDH) | This study |
| *E. coli* W3110△4-P_(L)_CAI_(L)_S | *E. coli* W3110△*ldhA*△*poxB*△*pflB*△*pta-ackA*△*frdBC*△*fumB*△*fumAC*  (pETM6R1-_(RBS03)_*Af*PYC-*Ec*CS-*Ec*ACN-_(RBS03)_*Ec*ICL-*Ec*SDH) | This study |
| *E. coli* W3110△4-P_(L)_CAI_(M)_S | *E. coli* W3110△*ldhA*△*poxB*△*pflB*△*pta-ackA*△*frdBC*△*fumB*△*fumAC*  (pETM6R1-_(RBS03)_*Af*PYC-*Ec*CS-*Ec*ACN-_(RBS09)_*Ec*ICL-*Ec*SDH) | This study |
| *E. coli* W3110△4-P_(L)_CAI_(H)_S | *E. coli* W3110△*ldhA*△*poxB*△*pflB*△*pta-ackA*△*frdBC*△*fumB*△*fumAC*  (pETM6R1-_(RBS03)_*Af*PYC-*Ec*CS-*Ec*ACN-_(RBS10)_*Ec*ICL-*Ec*SDH) | This study |
| *E. coli* W3110△4-P_(M)_CAI_(L)_S | *E. coli* W3110△*ldhA*△*poxB*△*pflB*△*pta-ackA*△*frdBC*△*fumB*△*fumAC*  (pETM6R1-_(RBS09)_*Af*PYC-*Ec*CS-*Ec*ACN-_(RBS03)_*Ec*ICL-*Ec*SDH) | This study |
| *E. coli* W3110△4-P_(M)_CAI_(M)_S | *E. coli* W3110△*ldhA*△*poxB*△*pflB*△*pta-ackA*△*frdBC*△*fumB*△*fumAC*  (pETM6R1-_(RBS09)_*Af*PYC-*Ec*CS-*Ec*ACN-_(RBS09)_*Ec*ICL-*Ec*SDH) | This study |
| *E. coli* W3110△4-P_(M)_CAI_(H)_S | *E. coli* W3110△*ldhA*△*poxB*△*pflB*△*pta-ackA*△*frdBC*△*fumB*△*fumAC*  (pETM6R1-_(RBS09)_*Af*PYC-*Ec*CS-*Ec*ACN-_(RBS10)_*Ec*ICL-*Ec*SDH) | This study |
| *E. coli* W3110△4-P_(H)_CAI_(L)_S | *E. coli* W3110△*ldhA*△*poxB*△*pflB*△*pta-ackA*△*frdBC*△*fumB*△*fumAC*  (pETM6R1-_(RBS10)_*Af*PYC-*Ec*CS-*Ec*ACN-_(RBS03)_*Ec*ICL-*Ec*SDH) | This study |
| *E. coli* W3110△4-P_(H)_CAI_(M)_S | *E. coli* W3110△*ldhA*△*poxB*△*pflB*△*pta-ackA*△*frdBC*△*fumB*△*fumAC*  (pETM6R1-_(RBS10)_*Af*PYC-*Ec*CS-*Ec*ACN-_(RBS09)_*Ec*ICL-*Ec*SDH) | This study |
| *E. coli* W3110△4-P_(H)_CAI_(H)_S | *E. coli* W3110△*ldhA*△*poxB*△*pflB*△*pta-ackA*△*frdBC*△*fumB*△*fumAC*  (pETM6R1-_(RBS10)_*Af*PYC-*Ec*CS-*Ec*ACN-_(RBS10)_*Ec*ICL-*Ec*SDH) | This study |
| *E. coli* W3110△4-P_(H)_CAI_(H)_SB | *E. coli* W3110△*ldhA*△*poxB*△*pflB*△*pta-ackA*△*frdBC*△*fumB*△*fumAC*  (pETM6R1-_(RBS10)_*Af*PYC-*Ec*CS-*Ec*ACN-_(RBS10)_*Ec*ICL-*Ec*SDH-dcuB) | This study |
| *E. coli* W3110△4-P_(H)_CAI_(H)_SC | *E. coli* W3110△*ldhA*△*poxB*△*pflB*△*pta-ackA*△*frdBC*△*fumB*△*fumAC*  (pETM6R1-_(RBS10)_*Af*PYC-*Ec*CS-*Ec*ACN-_(RBS10)_*Ec*ICL-*Ec*SDH-dcuC) | This study |
| *E. coli* W3110△4-P_(H)_CAI_(H)_SBC | *E. coli* W3110△*ldhA*△*poxB*△*pflB*△*pta-ackA*△*frdBC*△*fumB*△*fumAC*  (pETM6R1-_(RBS10)_*Af*PYC-*Ec*CS-*Ec*ACN-_(RBS10)_*Ec*ICL-*Ec*SDH-dcuBC) | This study |
| **Plasmids** |  |  |
| pKD3 | R6Kγ ori, Cm^R^, rgnB(Ter) | Invitrogen |
| pKD4 | R6Kγ ori, Km^R^, rgnB(Ter) | Invitrogen |
| pKD46 | R101 ori, Amp^R^, araBp-gam-bet-exo, repA101(ts) | Invitrogen |
| pCP20 | Amp^R^, Cm^R^, FLP recombinance | Invitrogen |
| pETM6R1 | ColE1 ori, Amp^R^, P_Trc_ |  |
| pTrcHisA-*Af*PYC | pTrcHisA with *pyc* gene from *Aspergillus flavus* |  |
| pET28a-*Ec*CS | pET28a with *gltA* gene from *E. coli* |  |
| pET28a-*Ec*ACN | pET28a with *acnB* gene from *E.coli* |  |
| pET28a-*Ec*ICL | pET28a with *aceA* gene from *E.coli* |  |
| pETM6R1-*Af*PYC | ColE1 ori, Amp^R^, P_Trc_-*Af*PYC | This study |
| pETM6R1-*Af*PYC-*Ec*CS | ColE1 ori, Amp^R^, P_Trc_-*Af*PYC, P_Trc_-*Ec*CS | This study |
| pETM6R1-*Af*PYC-*Ec*CS-*Ec*ACN | ColE1 ori, Amp^R^, P_Trc_-*Af*PYC, P_Trc_-*Ec*CS, P_Trc_-*Ec*ACN | This study |
| pETM6R1-*Af*PYC-*Ec*CS-*Ec*ACN-*Ec*ICL | ColE1 ori, Amp^R^, P_Trc_-*Af*PYC, P_Trc_-*Ec*CS, P_Trc_-*Ec*ACN, P_Trc_-*Ec*ICL | This study |
| pETM6R1-*Af*PYC-*Ec*CS-*Ec*ACN-*Ec*ICL  -*Ec*SDH | ColE1 ori, Amp^R^, P_Trc_-*Af*PYC, P_Trc_-*Ec*CS, P_Trc_-*Ec*ACN, P_Trc_-*Ec*ICL, P_Trc_-*Ec*SDH | This study |
| pETM6R1-_(RBS03)_*Af*PYC-*Ec*CS-*Ec*ACN  -*Ec*ICL-*Ec*SDH | ColE1 ori, Amp^R^, P_Trc_-_(RBS03)_*Af*PYC, P_Trc_-*Ec*CS, P_Trc_-*Ec*ACN, P_Trc_-*Ec*ICL, P_Trc_-*Ec*SDH | This study |
| pETM6R1-_(RBS09)_*Af*PYC-*Ec*CS-*Ec*ACN  -*Ec*ICL-*Ec*SDH | ColE1 ori, Amp^R^, P_Trc_-_(RBS09)_*Af*PYC, P_Trc_-*Ec*CS, P_Trc_-*Ec*ACN, P_Trc_-*Ec*ICL, P_Trc_-*Ec*SDH | This study |
| pETM6R1-_(RBS10)_*Af*PYC-*Ec*CS-*Ec*ACN  -*Ec*ICL-*Ec*SDH | ColE1 ori, Amp^R^, P_Trc_-_(RBS10)_*Af*PYC, P_Trc_-*Ec*CS, P_Trc_-*Ec*ACN, P_Trc_-*Ec*ICL, P_Trc_-*Ec*SDH | This study |
| pETM6R1-*Af*PYC-_(RBS03)_*Ec*CS-*Ec*ACN  -*Ec*ICL-*Ec*SDH | ColE1 ori, Amp^R^, P_Trc_-*Af*PYC, P_Trc_-_(RBS03)_*Ec*CS, P_Trc_-*Ec*ACN, P_Trc_-*Ec*ICL, P_Trc_-*Ec*SDH | This study |
| pETM6R1-*Af*PYC-_(RBS09)_*Ec*CS-*Ec*ACN  -*Ec*ICL-*Ec*SDH | ColE1 ori, Amp^R^, P_Trc_-*Af*PYC, P_Trc_-_(RBS09)_*Ec*CS, P_Trc_-*Ec*ACN, P_Trc_-*Ec*ICL, P_Trc_-*Ec*SDH | This study |
| pETM6R1-*Af*PYC-_(RBS10)_*Ec*CS-*Ec*ACN  -*Ec*ICL-*Ec*SDH | ColE1 ori, Amp^R^, P_Trc_-*Af*PYC, P_Trc_-_(RBS10)_*Ec*CS, P_Trc_-*Ec*ACN, P_Trc_-*Ec*ICL, P_Trc_-*Ec*SDH | This study |
| pETM6R1-*Af*PYC-*Ec*CS-_(RBS03)_*Ec*ACN  -*Ec*ICL-*Ec*SDH | ColE1 ori, Amp^R^, P_Trc_-*Af*PYC, P_Trc_-*Ec*CS, P_Trc_-_(RBS03)_*Ec*ACN, P_Trc_-*Ec*ICL, P_Trc_-*Ec*SDH | This study |
| pETM6R1-*Af*PYC-*Ec*CS-_(RBS09)_*Ec*ACN  -*Ec*ICL-*Ec*SDH | ColE1 ori, Amp^R^, P_Trc_-*Af*PYC, P_Trc_-*Ec*CS, P_Trc_-_(RBS09)_*Ec*ACN, P_Trc_-*Ec*ICL, P_Trc_-*Ec*SDH | This study |
| pETM6R1-*Af*PYC-*Ec*CS-_(RBS10)_*Ec*ACN  -*Ec*ICL-*Ec*SDH | ColE1 ori, Amp^R^, P_Trc_-*Af*PYC, P_Trc_-*Ec*CS, P_Trc_-_(RBS10)_*Ec*ACN, P_Trc_-*Ec*ICL, P_Trc_-*Ec*SDH | This study |
| pETM6R1-*Af*PYC-*Ec*CS-*Ec*ACN  -_(RBS03)_*Ec*ICL-*Ec*SDH | ColE1 ori, Amp^R^, P_Trc_-*Af*PYC, P_Trc_-*Ec*CS, P_Trc_-*Ec*ACN, P_Trc_-_(RBS03)_*Ec*ICL, P_Trc_-*Ec*SDH | This study |
| pETM6R1-*Af*PYC-*Ec*CS-*Ec*ACN  -_(RBS09)_*Ec*ICL-*Ec*SDH | ColE1 ori, Amp^R^, P_Trc_-*Af*PYC, P_Trc_-*Ec*CS, P_Trc_-*Ec*ACN, P_Trc_-_(RBS09)_*Ec*ICL, P_Trc_-*Ec*SDH | This study |
| pETM6R1-*Af*PYC-*Ec*CS-*Ec*ACN  -_(RBS10)_*Ec*ICL-*Ec*SDH | ColE1 ori, Amp^R^, P_Trc_-*Af*PYC, P_Trc_-*Ec*CS, P_Trc_-*Ec*ACN, P_Trc_-_(RBS10)_*Ec*ICL, P_Trc_-*Ec*SDH | This study |
| pETM6R1-*Af*PYC-*Ec*CS-*Ec*ACN  -*Ec*ICL-_(RBS03)_*Ec*SDH | ColE1 ori, Amp^R^, P_Trc_-*Af*PYC, P_Trc_-*Ec*CS, P_Trc_-*Ec*ACN, P_Trc_-*Ec*ICL, P_Trc_-_(RBS03)_*Ec*SDH | This study |
| pETM6R1-*Af*PYC-*Ec*CS-*Ec*ACN  -*Ec*ICL-_(RBS09)_*Ec*SDH | ColE1 ori, Amp^R^, P_Trc_-*Af*PYC, P_Trc_-*Ec*CS, P_Trc_-*Ec*ACN, P_Trc_-*Ec*ICL, P_Trc_-_(RBS09)_*Ec*SDH | This study |
| pETM6R1-*Af*PYC-*Ec*CS-*Ec*ACN  -*Ec*ICL-_(RBS10)_*Ec*SDH | ColE1 ori, Amp^R^, P_Trc_-*Af*PYC, P_Trc_-*Ec*CS, P_Trc_-*Ec*ACN, P_Trc_-*Ec*ICL, P_Trc_-_(RBS10)_*Ec*SDH | This study |
| pETM6R1-_(RBS03)_*Af*PYC-*Ec*CS-*Ec*ACN  -_(RBS03)_*Ec*ICL-*Ec*SDH | ColE1 ori, Amp^R^, P_Trc_-_(RBS03)_*Af*PYC, P_Trc_-*Ec*CS, P_Trc_-*Ec*ACN, P_Trc_-_(RBS03)_*Ec*ICL, P_Trc_-*Ec*SDH | This study |
| pETM6R1-_(RBS03)_*Af*PYC-*Ec*CS-*Ec*ACN  -_(RBS09)_*Ec*ICL-*Ec*SDH | ColE1 ori, Amp^R^, P_Trc_-_(RBS03)_*Af*PYC, P_Trc_-*Ec*CS, P_Trc_-*Ec*ACN, P_Trc_-_(RBS09)_*Ec*ICL, P_Trc_-*Ec*SDH | This study |
| pETM6R1-_(RBS03)_*Af*PYC-*Ec*CS-*Ec*ACN  -_(RBS10)_*Ec*ICL-*Ec*SDH | ColE1 ori, Amp^R^, P_Trc_-_(RBS03)_*Af*PYC, P_Trc_-*Ec*CS, P_Trc_-*Ec*ACN, P_Trc_-_(RBS10)_*Ec*ICL, P_Trc_-*Ec*SDH | This study |
| pETM6R1-_(RBS09)_*Af*PYC-*Ec*CS-*Ec*ACN  -_(RBS03)_*Ec*ICL-*Ec*SDH | ColE1 ori, Amp^R^, P_Trc_-_(RBS09)_*Af*PYC, P_Trc_-*Ec*CS, P_Trc_-*Ec*ACN, P_Trc_-_(RBS03)_*Ec*ICL, P_Trc_-*Ec*SDH | This study |
| pETM6R1-_(RBS09)_*Af*PYC-*Ec*CS-*Ec*ACN  -_(RBS09)_*Ec*ICL-*Ec*SDH | ColE1 ori, Amp^R^, P_Trc_-_(RBS09)_*Af*PYC, P_Trc_-*Ec*CS, P_Trc_-*Ec*ACN, P_Trc_-_(RBS09)_*Ec*ICL, P_Trc_-*Ec*SDH | This study |
| pETM6R1-_(RBS09)_*Af*PYC-*Ec*CS-*Ec*ACN  -_(RBS10)_*Ec*ICL-*Ec*SDH | ColE1 ori, Amp^R^, P_Trc_-_(RBS09)_*Af*PYC, P_Trc_-*Ec*CS, P_Trc_-*Ec*ACN, P_Trc_-_(RBS10)_*Ec*ICL, P_Trc_-*Ec*SDH | This study |
| pETM6R1-_(RBS10)_*Af*PYC-*Ec*CS-*Ec*ACN  -_(RBS03)_*Ec*ICL-*Ec*SDH | ColE1 ori, Amp^R^, P_Trc_-_(RBS10)_*Af*PYC, P_Trc_-*Ec*CS, P_Trc_-*Ec*ACN, P_Trc_-_(RBS03)_*Ec*ICL, P_Trc_-*Ec*SDH | This study |
| pETM6R1-_(RBS10)_*Af*PYC-*Ec*CS-*Ec*ACN  -_(RBS09)_*Ec*ICL-*Ec*SDH | ColE1 ori, Amp^R^, P_Trc_-_(RBS10)_*Af*PYC, P_Trc_-*Ec*CS, P_Trc_-*Ec*ACN, P_Trc_-_(RBS09)_*Ec*ICL, P_Trc_-*Ec*SDH | This study |
| pETM6R1-_(RBS10)_*Af*PYC-*Ec*CS-*Ec*ACN  -_(RBS10)_*Ec*ICL-*Ec*SDH | ColE1 ori, Amp^R^, P_Trc_-_(RBS10)_*Af*PYC, P_Trc_-*Ec*CS, P_Trc_-*Ec*ACN, P_Trc_-_(RBS10)_*Ec*ICL, P_Trc_-*Ec*SDH | This study |
| pETM6R1-_(RBS10)_*Af*PYC-*Ec*CS-*Ec*ACN  -_(RBS10)_*Ec*ICL-*Ec*SDH-dcuB | ColE1 ori, Amp^R^, P_Trc_-_(RBS10)_*Af*PYC, P_Trc_-*Ec*CS, P_Trc_-*Ec*ACN, P_Trc_-_(RBS10)_*Ec*ICL, P_Trc_-*Ec*SDH, P_Trc_-dcuB | This study |
| pETM6R1-_(RBS10)_*Af*PYC-*Ec*CS-*Ec*ACN  -_(RBS10)_*Ec*ICL-*Ec*SDH-dcuC | ColE1 ori, Amp^R^, P_Trc_-_(RBS10)_*Af*PYC, P_Trc_-*Ec*CS, P_Trc_-*Ec*ACN, P_Trc_-_(RBS10)_*Ec*ICL, P_Trc_-*Ec*SDH, P_Trc_-dcuC | This study |
| pETM6R1-_(RBS10)_*Af*PYC-*Ec*CS-*Ec*ACN  -_(RBS10)_*Ec*ICL-*Ec*SDH-dcuBC | ColE1 ori, Amp^R^, P_Trc_-_(RBS10)_*Af*PYC, P_Trc_-*Ec*CS, P_Trc_-*Ec*ACN, P_Trc_-_(RBS10)_*Ec*ICL, P_Trc_-*Ec*SDH, P_Trc_-dcuBC | This study |

**References**

1. Dong X, Chen X, Qian Y, Wang Y, Wang L, Qiao W, Liu L: Metabolic engineering of *Escherichia coli* W3110 to produce L-malate. *Biotechnol Bioeng* 2017, 114:656-664.

2. Zhang Q, Yao R, Chen X, Liu L, Xu S, Chen J, Wu J: Enhancing fructosylated chondroitin production in *Escherichia coli* K4 by balancing the UDP-precursors. *Metab Eng* 2018, 47:314-322.

3. Gao C, Wang S, Hu G, Guo L, Chen X, Xu P, Liu L: Engineering *Escherichia coli* for malate production by integrating modular pathway characterization with CRISPRi-guided multiplexed metabolic tuning. *Biotechnol Bioeng* 2018, 115:661-672.
